# Complete mitochondrial genome of the Australian basket clam *Corbicula australis*, an exotic species found in New Zealand

**DOI:** 10.1128/mra.00084-26

**Published:** 2026-06-24

**Authors:** Ruy Jauregui, Yee Syuen Low, Maeve Ballantyne, Susana Enriquez, Jonathan Foxwell, Stefan D. M. Maday, Andrew Wilson, Andrea Barcelo, Rachel Hooks, Helen Johnston, Michelle McCulley

**Affiliations:** 1 Animal Health Laboratory, Ministry for Primary Industries, Upper Hutt, New Zealand; 2 Aquatic Health, Ministry for Primary Industries, Upper Hutt, New Zealand; University of California Riverside, Riverside, California, USA

**Keywords:** mitochondria, clam, genomes

## Abstract

Freshwater clams belonging to the genus *Corbicula* have been identified as exotic animals in New Zealand. Here, we present the complete mitochondrial genome of *C. australis* from a sample collected in the North Island of New Zealand.

## ANNOUNCEMENT

*Corbicula australis* (Cyrenidae; Bivalvia), a bivalve mollusk endemic to Australia, has been declared as an exotic, unwanted organism in New Zealand, as described in the Official New Zealand Pest Register (https://pierpestregister.mpi.govt.nz/pests-of-concern/pest-details/?id=108264). Availability of genomic data for this organism is scant, and expanding genomic resources will strengthen our identification and diagnostic methods.

Samples from an exotic clam were collected from a small man-made lagoon in the Taupō region in the North Island of New Zealand (−38.6285, 176.0974) in March 2024. Sampling permits are covered under the New Zealand Biosecurity Act 1993. Morphological analysis and genetic analysis of the 28S (99.1% identity to KM598278.1), 16S (99.8% identity to AF152023.1), and COX1 (99.8% identity to AF196274.1) genes identified clams as *C. australis* (internal communication). Whole genome shotgun sequencing provided the data used to obtain a complete assembly of its mitogenome.

Adductor muscle tissue was sliced with a sterile scalpel, and a fragment of approximately 20 mg was used for DNA extraction with a QIAamp DNA Mini Kit (Qiagen, Germany). DNA concentration was quantified using the Qubit 1× dsDNA High Sensitivity Assay Kit (Invitrogen, Thermo Fisher Scientific, Waltham, MA, USA) and measured on a Qubit 3.0 Fluorometer (Invitrogen, Thermo Fisher Scientific, Waltham, MA, USA). DNA was fragmented to uniform length using a Covaris g-tube (Covaris Inc., Woburn, MA, USA); 55 µL of DNA was loaded into the Covaris g-tube and centrifuged at 8,000 rpm for 1 min on both sides of the tube. The DNA size was determined using the genomic DNA ScreenTape Assay (Agilent Technologies, Santa Clara, CA, USA) on the Agilent TapeStation 4200 system (Agilent Technologies, Santa Clara, CA, USA) before and after fragmentation. Post-fragmentation fragments had an average size of 12,104 bp.

Fragmented DNA was purified using a 1.0× ratio of AMPure XP Beads (Beckman Coulter, Brea, CA, USA) to remove contaminants in the sample and quantified as above.

The sample was processed using the Oxford Nanopore Technology (ONT) Ligation Sequencing Kit V14 (SQK-LSK114) (Oxford Nanopore Technologies, London, UK). The library was loaded onto a PromethION flow-cell R 10.4.1 (Oxford Nanopore Technologies, London, UK) and sequenced on a P2-Solo till the end of life of the pores.

Basecalling was performed by Dorado v0.9.6 (https://github.com/nanoporetech/dorado) with the high-accuracy model DNAv5.2.0. Bases with Q < 10 were filtered during sequencing. Fastq records were compiled into a single file before mapping.

Reads were mapped to the *C. fluminea* mitogenome OR521035 using Minimap2 v2.28 (1) with the “-ax lr:hq” option. Mapped reads were used to generate a consensus sequence using SAMtools v1.22 (2) and circularized using Geneious Prime. The genome was polished using Medaka v1.11.1 (https://github.com/nanoporetech/medaka) and annotated using MITOS2 v2.1.0 (3) with the invertebrates genetic code table. Default parameters were used except where otherwise noted.

**Fig 1:**
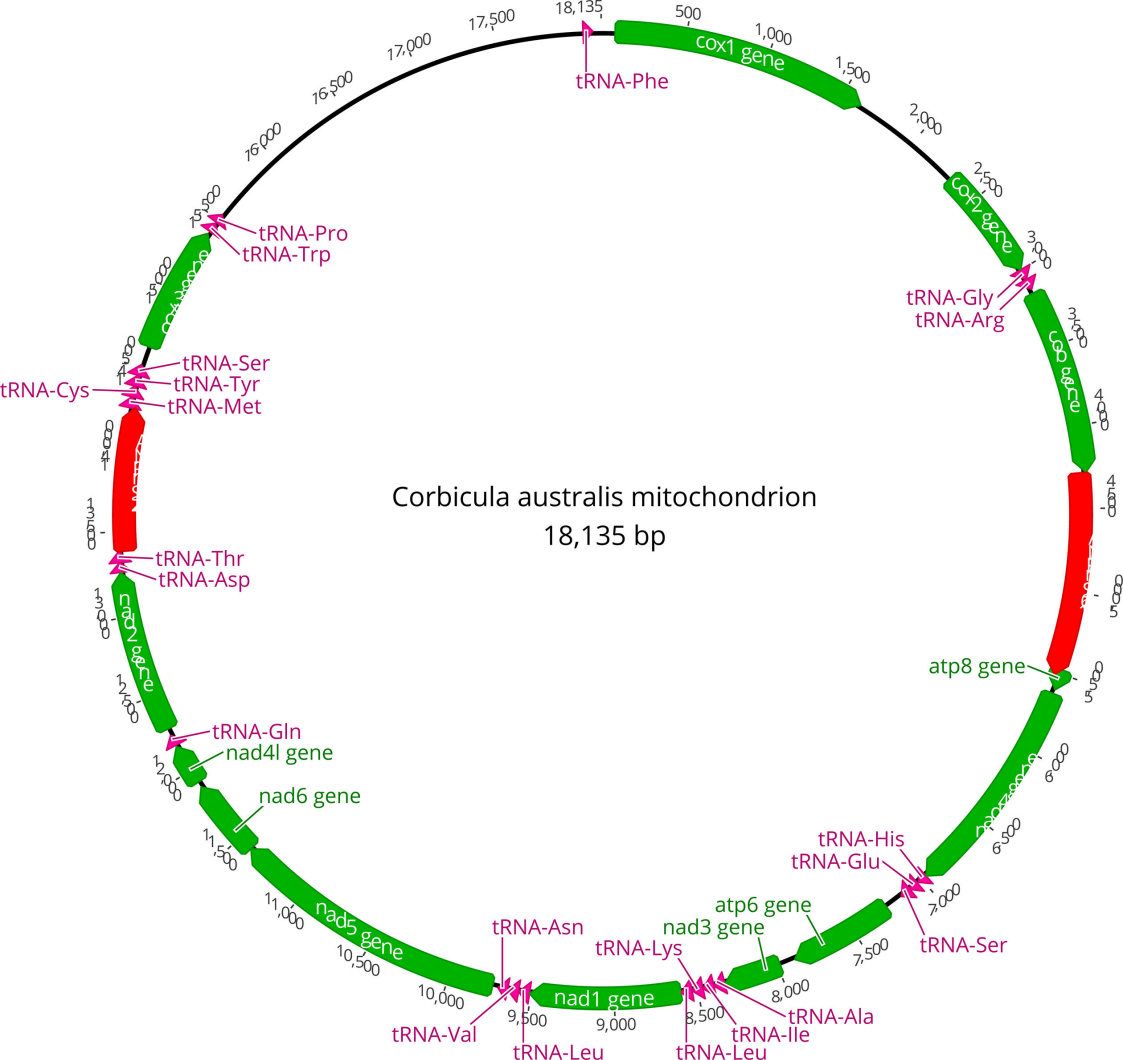
Visual representation of the *C. australis* mitogenome.

A total of 41,336 reads were mapped, with an N50 of 5.514 Kbp, and a mean coverage of 9,022×. The assembled genome consists of a single contiguous fragment of 18,135 nt. with GC% of 30.56, encoding 13 proteins, 2 rRNAs, and 22 tRNAs. The genome is complete when compared to the *C. fluminea* mitogenome. Figure 1, representing the sequence genome, was generated with Geneious Prime 2025.1.3 (www.geneious.com).

## Data Availability

The completed genome has been submitted to GenBank under accession no. PX857320. The unassembled reads have been deposited in the SRA archive under accession nos. SRR36670343 (mitochondrial reads) and SRR38514866 (complete read set).
